# Increased HIV‐1 infection in PBMCs treated in vitro with menstrual cycle phase hormones or medroxyprogesterone acetate likely occurs via different mechanisms

**DOI:** 10.1111/aji.13643

**Published:** 2022-11-02

**Authors:** Alexis J. Bick, Chanel Avenant, Michele Tomasicchio, Zephne van der Spuy, Janet P. Hapgood

**Affiliations:** ^1^ Department of Molecular and Cell Biology University of Cape Cape Town South Africa; ^2^ Centre for Lung Infection and Immunity, Division of Pulmonology, Department of Medicine University of Cape Town and UCT Lung Institute Cape Town South Africa; ^3^ South African MRC Centre for the Study of Antimicrobial Resistance University of Cape Town Cape Town South Africa; ^4^ Department of Obstetrics and Gynaecology University of Cape Town, Groote Schuur Hospital Cape Town South Africa; ^5^ Institute of Infectious Disease and Molecular Medicine University of Cape Town Cape Town South Africa

**Keywords:** CCR5, glucocorticoid receptor, HIV‐1, luteal phase, medroxyprogesterone acetate, menstrual cycle, PBMCs

## Abstract

**Problem:**

Both luteal phase progesterone (P4) levels and use of the intramuscular (IM) injectable progestin‐only contraceptive depo‐medroxyprogesterone acetate (DMPA‐IM) have been linked to increased S/HIV acquisition in animal, clinical and in vitro models. Several plausible mechanisms could explain MPA‐induced HIV‐1 acquisition while those for the luteal phase are underexplored.

**Method of study:**

Peripheral blood mononuclear cells (PBMCs) were treated with P4 and estrogen at concentrations mimicking the luteal phase, follicular phase or with levels of MPA mimicking peak serum levels in DMPA‐IM users. Cells were infected with an R5‐tropic infectious molecular clone and HIV‐1 infection was measured. A role for the glucocorticoid receptor (GR) was investigated using the GR/PR antagonist RU486. CCR5 protein levels and activation status, assessed by levels of the activation marker CD69, were measured by flow cytometry after treatment in vitro and in PBMCs from naturally‐cycling women or DMPA‐IM users.

**Results:**

Both MPA and luteal phase hormones significantly increased HIV‐1 infection in vitro. However, MPA but not luteal phase hormones increased the CD4+/CD8+ T cell ratio, CCR5 protein expression on CD4+ T cells and increased expression of the activation marker CD69. The GR is involved in MPA‐induced, but not luteal phase hormone‐induced increased HIV‐1 infection. In DMPA‐IM users, the frequency of CCR5‐expressing CD3+ and CD8+ cells was higher than for women in the luteal phase.

**Conclusions:**

MPA increases HIV‐1 infection in a manner different from that of luteal phase hormones, most likely involving the GR and at least in part changes in the frequency and/or expression of CCR5 and CD69.

## 1 INTRODUCTION

Differential susceptibility to HIV‐1 infection during the menstrual cycle or with injectable contraceptive use is a major concern for women's health. The menstrual cycle is characterized by fluctuations in female sex hormones, including estrogen (E2), progesterone (P4), luteinizing hormone (LH) and follicle‐stimulating hormone (FSH). During the P4‐dominant luteal phase, there is a hypothesized “window of vulnerability” for infection, in which sex hormones suppress immunity to support fertilization and implantation.^1^, ^2^, ^3^, ^4^, ^5^ Consistent with this hypothesis are findings in non‐human primate models showing enhanced susceptibility to SIV/SHIV infection during the luteal phase.^6^, ^7^, ^8^, ^9^, ^10^, ^11^, ^12^, ^13^ Furthermore, some in vitro models have suggested higher productive HIV‐1 infection during the luteal compared to the follicular phase in cervical explant tissue,^10^ although other reports show no difference in ex vivo HIV‐1 infection of cervical explant tissue between luteal and follicular phases^14^, ^15^ and one study showed that treatment with follicular phase hormones increased HIV‐1 replication in PBMCs.^16^ Recent evidence suggests that women in the luteal phase possess higher frequencies of cervical HIV‐1 target cells compared to women in the follicular phase,^17^, ^18^ including cells expressing the HIV‐1 co‐receptor CCR5.^17^ Recent studies using RNASeq in cervical tissue and proteomics in cervicovaginal lavage samples indicate that women in the luteal phase exhibit signatures associated with increased HIV‐1 susceptibility.^19^, ^20^ Together, these studies suggest that increased HIV‐1 acquisition occurs in women during the luteal compared to the follicular phase.

The high levels of P4 are suggested to account for differences between the menstrual cycle phases. Serum P4 concentrations range from .4 to 1.6 nM during the follicular phase to a wide range of 1–86 nM during the luteal phase,^15^, ^21^, ^22^, ^23^ while that of E2 ranges from 50 to 1500 pM during the follicular phase and 50–1100 pM across the luteal phase.^23^ Consistent with the proposed role of higher P4 in the luteal phase in SIV/HIV‐1 susceptibility, are the findings of increased SIV susceptibility in macaques given a subcutaneous P4‐releasing capsule^24^ and increased HIV‐1 infection in activated PBMCs treated with P4,^25^, ^26^, ^27^ although P4 treatment inhibited HIV‐1 infection of PBMCs in vitro in one study^28^ and in placental trophoblasts in another study.^29^ However, pregnant women with P4 levels of up to 1 μM^30^ have an increased susceptibility to HIV‐1 infection,^31^, ^32^ possibly due to higher PBMC CCR5 levels.^33^

Depo‐medroxyprogesterone acetate‐intramuscular (DMPA‐IM), containing the synthetic progestogen MPA, has also been linked to increased HIV‐1 acquisition risk in multiple clinical observational,^34^, ^35^, ^36^ animal^37^, ^38^, ^39^ and in vitro studies.^25^, ^40^, ^41^, ^42^, ^43^ Whether DMPA‐IM increases HIV‐1 infection in vivo is controversial. Some observational studies showed no significant increased infection in DMPA‐IM users^44^, ^45^, ^46^, ^47^ and the recent ECHO trial did not detect a difference greater than 50% in infection for three contraceptives (DMPA‐IM, levonorgestrel implant, Cu‐IUD) relative to each other.^48^ In DMPA‐IM users, the average detected peak MPA serum levels are approximately 5–40 nM and the maximum levels reported reach up to 65–100 nM.^49^, ^50^, ^51^, ^52^ Multiple plausible biological mechanisms have been suggested to explain how DMPA‐IM can increase HIV‐1 infection in the female reproductive tract (FRT) and in peripheral blood.^50^, ^53^ These include changes in barrier function, adaptive immunity (frequency of key immune cell types and their activation status) and increased levels of the CCR5 co‐receptor, which would facilitate founder R5‐tropic viral entry.^50^, ^54^, ^55^

Importantly, while MPA and P4 act via the progesterone receptor (PR), MPA also binds to the glucocorticoid receptor (GR) with relatively high affinity,^56^, ^57^, ^58^ and regulates GR target genes.^59^ Since the GR is a key regulator of immune function, DMPA‐IM could impact on susceptibility to infection in women via the GR. MPA can act as a potent partial to full agonist for the GR,^59^ depending on the cellular context, while P4 is a partial agonist with relatively low GR affinity and potency,^57^, ^58^ but with immunosuppressive effects at high concentrations during pregnancy.^60^, ^61^, ^62^ While changes in barrier function by both P4 and DMPA‐IM are likely mediated by the PR,^63^ whether differential effects on immune function and HIV‐1 susceptibility occur via the GR for luteal phase P4 versus DMPA‐IM is unknown.

Here we explore potential biological and immunological mechanisms by which MPA and luteal phase hormones increase HIV‐1 acquisition in PBMCs, including GR requirement, CCR5 levels and immune cell activation.

## 2 METHODS

### 2.1 Cell culture and hormone treatment

HEK293T cells (America Type Culture Collection, USA) were cultured as described previously^40^ and routinely checked for mycoplasma contamination.^64^

Human PBMC use was approved by the Human Research Ethics Committee at the University of Cape Town (study HREC 210/2011). UCT Faculty biosafety approvals (BSC004_2018) were obtained for the current study. Buffy packs from healthy female HIV‐negative donors of unknown menstrual cycle phase were obtained from the Western Cape Blood Services for in vitro treatments. PBMCs from these buffy packs (blood bank PBMCs or bbPBMCs) were isolated from whole blood as previously described^65^ and frozen at ‐80°C in full high‐glucose (4.5 g/ml) RPMI medium (Lonza, Switzerland) with 10% DMSO and 40% charcoal‐stripped (cs)‐FCS until use. Additional archived PBMCs were obtained from a previously‐described study where cervical explants and matched blood samples were obtained from HIV‐negative women undergoing hysterectomies for benign reasons^41^ (PBMCs from women having hysterectomies or hPBMCs). hPBMCs were isolated and frozen as above. Menstrual cycle phase was determined by levels of LH, FSH, E2 and P4 within the ranges established by the South African National Health Laboratory Services (NHLS),^41^ for at least three of the menstrual cycle hormones. LH, FSH, E2, and P4 were measured in serum by electrochemiluminescence assays on the Roche Cobas e601. DMPA‐IM users were determined by self‐reported last dose of Depo Provera or Petogen within the previous 3 months (Supplementary Table S1), but serum MPA concentrations were not measured as serum was not collected. Age, HIV, HPV and HSV status for the cohort are recorded in Supplementary Table S1, and the clinical information is summarized in Supplementary Table S2.

bbPBMCs were cultured as previously described.^40^ bbPBMCs were treated with combinations of 17β‐estradiol (E2, E2758, Sigma Aldrich, South Africa) and 4‐pregnene‐3,20‐dione (progesterone; P4, P0130, Sigma Aldrich, South Africa) mimicking the luteal phase (10 nM P4 + 400 pM E2) or the follicular phase (1 nM P4 + 400 pM E2) of the menstrual cycle, within the ranges of reported menstrual cycle E2/P4 concentrations.^23^ Cells were also treated with 6α‐methyl‐17α‐hydroxy‐progesterone acetate (medroxyprogesterone acetate; MPA, M1629, Sigma Aldrich, South Africa) at 100 nM to represent the upper peak concentration of MPA in the serum of DMPA‐IM users.^49^, ^52^ For some experiments, cells were co‐treated with 100 nM of the GR/PR antagonist 11β‐(4‐dimethylamino)phenyl‐17β‐hydroxy‐17‐(1propynyl)estra‐4,9‐dien‐3‐one (Mifepristone; RU486, M8046, Sigma Aldrich, South Africa). All steroid hormones were prepared in absolute ethanol (EtOH) such that final EtOH concentration was .1% (v/v).

### 2.2 Flow cytometry

Non‐activated bbPBMCs seeded at 4 × 10^6^ were treated in vitro with the indicated ligands for 7 days in full RPMI containing 10% (v/v) cs‐FCS, 2 mM L‐glutamine, .1 mg/ml sodium pyruvate, 100 IU/ml penicillin, 100 mg/ml streptomycin and 30 U/ml IL‐2, while hPBMCs were thawed and rested overnight in full RPMI in an incubator at 37°C. Cells were washed with 1 X PBS containing 1% cs‐FCS and subsequently stained with anti‐human CD3 FITC (300306), anti‐CD4 PE‐DAZZLE 594 (357412), anti‐CD8 PE/Cy5 (300910), anti‐CD14 APC (325608), anti‐CCR5 PE (359106), anti‐CD69 PE/Cy7 (310912) and the viability dye, ZOMBIE NIR (423113) (Biolegend, USA), for 15 min in the dark at room temperature. Fluorescence minus‐one (FMO) controls were used for gating as indicated in Figure S1. Cells were washed with 1 X PBS containing 1% cs‐FCS then resuspended in 1 X Cell Fix solution (BD, USA) and analyzed using a LSRII flow cytometer (BD, USA) and FlowJo software version 10.1 (Treestar Inc., Ashland, Ore). Dead cells were excluded from the scatter plots prior to analysis and only single cellular populations were analyzed (Figure S1). Results were plotted as frequency (percentage of total), or expression as median fluorescence intensity (MFI) per number of double‐positive cells.

### 2.3 PBMC infection assays

Viral stocks of the R5‐tropic infectious molecular clone NL‐LucR. T2A‐BaL.ecto (HIV‐1_BaL‐Renilla_)^66^ were prepared as previously described.^40^, ^67^ Viral titres were determined as previously described^40^, ^66^ and calculated as log infectious units (IU)/ml.^68^

Non‐activated bbPBMCs were seeded at 4 × 10^6^ and treated in vitro with the indicated ligands for 2 or 7 days. bbPBMCs were then infected using 10 IU/ml HIV‐1_BaL‐Renilla_ or the equivalent volume of virus control for 2 h at 37°C, and subsequently washed three times in 1 X PBS containing 1% cs‐FCS, resuspended in hormone‐free full RPMI containing IL‐2 and incubated for a further 5 days. PBMCs were pelleted by centrifugation then harvested either for *Renilla* luciferase expression (infection) using *Renilla* luciferin according to the manufacturer's specifications (Promega, USA), measured on a luminometer (Modulus microplate, Turner Biosystems, USA) or for cell viability using the MTT assay, measured on a spectrophotometer (Thermo Scientific, USA) at 595 nm. Infection was calculated as RLU of each quadruplicate divided by the average absorbance at 595 nm of the four quadruplicate wells (RLU/ave MTT). Relative infection was determined by setting the vehicle control (EtOH) in the presence of virus to 100% or as indicated in the figure legend. Archived hPBMCs were not used for infection experiments due to low sample availability.

### 2.4 Protein isolation and western blotting

Total protein lysates from PBMCs were harvested using .1 M N‐[Tris(hydroxymethyl)‐methyl]‐3‐aminopropanesulfonic acid (TAPS) buffer, pH 9.5, supplemented with protease inhibitors (Roche Applied Science, South Africa) as previously described.^69^

Western blotting was carried out as previously described.^70^ Blots were probed with primary antibodies against GR (1:2000; H‐300, Santa Cruz Biotechnology), PR (1:500; NCL‐LPGR‐312, Leica Biosystems) and GAPDH (1:15000; 0411, Santa Cruz Biotechnology). Horseradish peroxidise‐conjugated secondary antibodies, anti‐mouse (1:4000; sc‐2005) and anti‐rabbit (1:10000; sc‐2313), were purchased from Santa Cruz Biotechnology, USA.

### 2.5 Statistical analysis

Statistical tests were carried out using GraphPad Prism software (version 7). Data were first analyzed for normal distribution using a D'Agostino and Pearson normality test (*n* ≥ 8) or a Shapiro Wilk normality test (*n* < 8). Normally distributed data were analyzed using either one‐way ANOVA with either Dunnett's (compared to control sample) or Tukey's (compared to every other sample) post tests for multiple comparisons or unpaired *t*‐tests for comparisons between two samples. Non‐normally distributed data were analyzed using a non‐parametric Kruskal‐Wallis test (non‐parametric one‐way ANOVA) with Dunn's post‐test (compared to control sample) for multiple comparisons or a Wilcoxon signed‐rank test for comparisons between two samples. HIV‐1 infection assays, where data were measured in quadruplicate, were analyzed using two‐way ANOVA with either Dunnett's or Tukey's post tests for multiple comparisons.

## 3 RESULTS

### 3.1 MPA and luteal phase E2+P4 increase HIV‐1 replication in bbPBMCs

**FIGURE 1:**
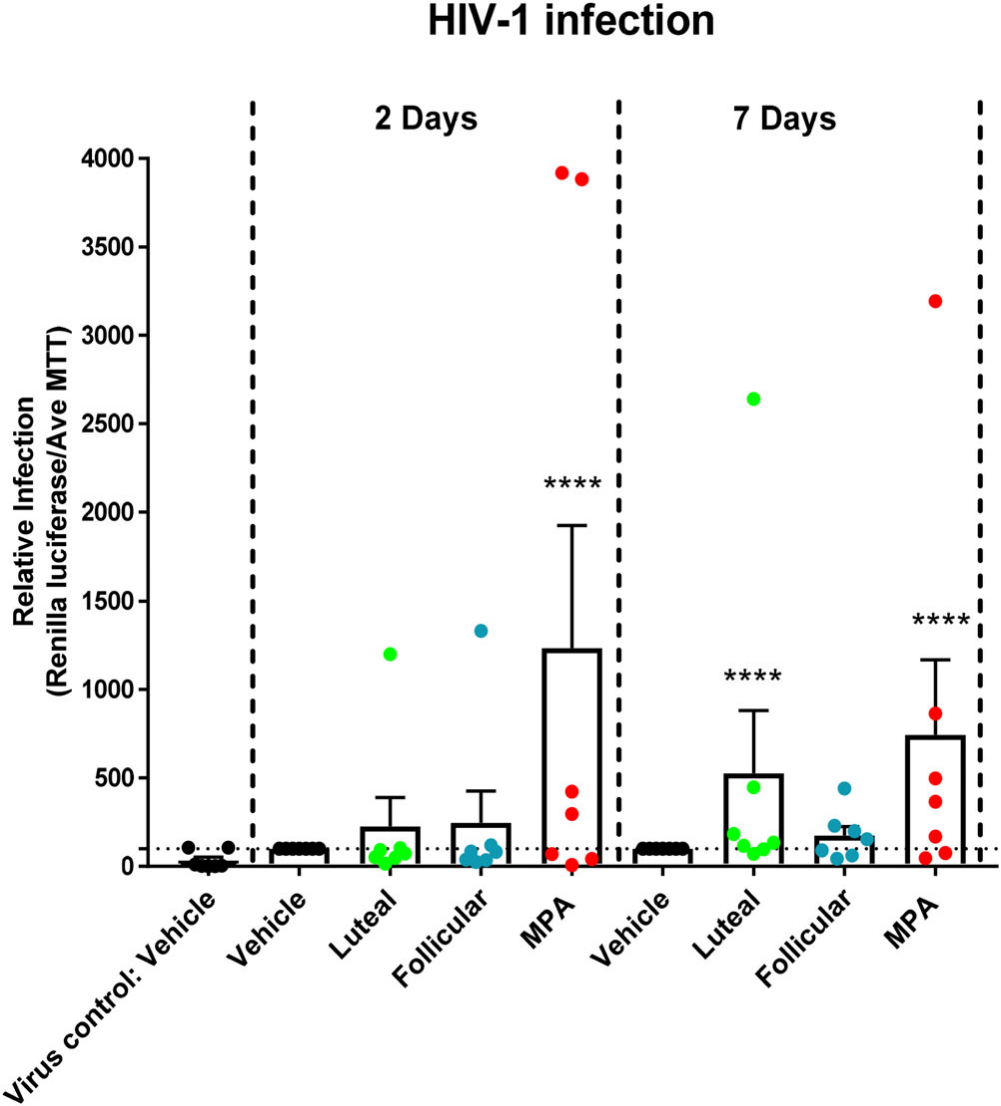
HIV‐1 replication in blood bank peripheral blood mononuclear cells (bbPBMCs) increases upon treatment with luteal phase estrogen (E2) + progesterone (P4) and medroxyprogesterone acetate (MPA). bbPBMCs were treated for 2 or 7 days, then infected with R5‐tropic HIV‐1 for 2 h. After 5 days in ligand‐free media, cells were harvested for *Renilla* luciferase (infection) or for cell viability (MTT). Pooled data are from at least three independent experiments, with two to three PBMC donors each, for a total of seven independent donors. Infection was plotted as mean + standard error of the mean (SEM) relative to each day's vehicle control in the presence of virus set to 100%. Statistical comparisons were carried out using a two‐way ANOVA with Dunnett's multiple comparisons post‐test. Stars above bars indicate significance compared to own vehicle control in the presence of virus, with **** indicating *p* < .0001

To investigate whether hormones representing the luteal phase or DMPA‐IM have direct effects on HIV‐1 infection in vitro, bbPBMCs were treated for 2 or 7 days with combinations of E2 and P4 representing the luteal (10 nM P4 + 400 pM E2) or follicular phase (1 nM P4 + 400 pM E2), or MPA (100 nM), and infected with HIV_BaL. A significant increase in *Renilla* luciferase expression was observed only in the presence of HIV_BaL and not with the hormones alone (Figure S2). MPA significantly increased HIV‐1 infection at both day 2 and day 7, while luteal phase E2+P4 significantly increased infection at day 7 (Figure 1) and at day 2 in some experiments (Figure S3). While P4 levels during the luteal phase can reach up to ∼70 nM,^23^ treatment with 100 nM P4 or 100 nM P4 + 400 pM E2 for 2 days did not further enhance HIV‐1 infection (Figure S3), and therefore 10 nM P4 + 400 pM E2 and a 7 day treatment was used to represent the luteal phase in subsequent experiments. Follicular phase E2+P4 did not change infection at either time point (Figure 1).

### 3.2 MPA‐ but not luteal phase E2+P4‐increased HIV‐1 replication most likely involves the GR

MPA and P4 exert their contraceptive actions via the PR. However, we do not detect PR mRNA or protein expression in PBMCs under basal conditions.^65^ While there is evidence showing that E2 can up‐regulate PR expression,^71^, ^72^, ^73^ treatment with the luteal phase combination of E2+P4 did not induce detectable PR protein expression in bbPBMCs (Figure S4). Our results suggest that the responses to these ligands in bbPBMCs under our conditions are therefore likely to occur via the GR and/or estrogen receptor (ER), which are endogenously expressed in PBMCs.^65^, ^74^

**FIGURE 2:**
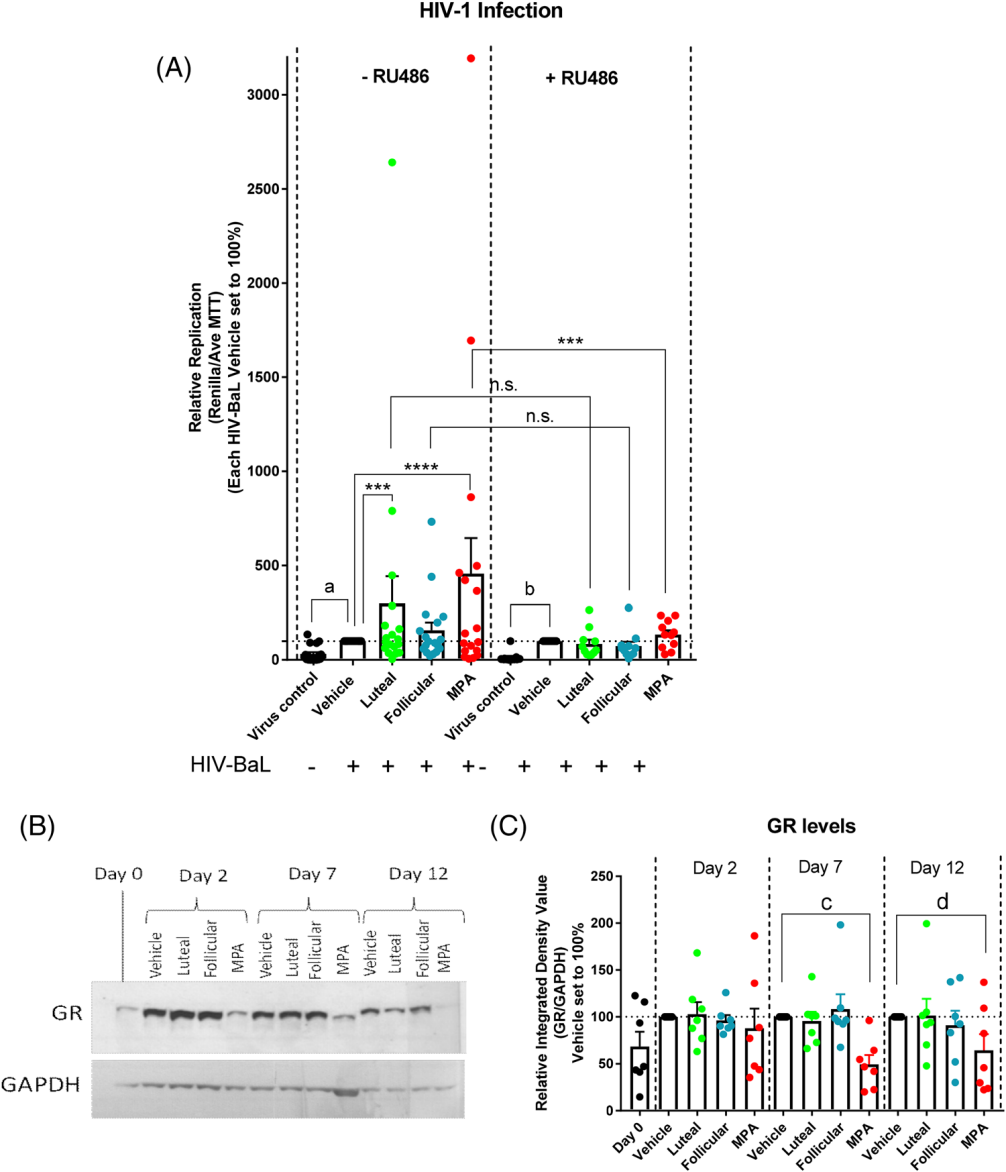
MPA‐increased but not luteal phase‐increased HIV‐1 infection is mediated by the glucocorticoid receptor (GR). (A) bbPBMCs were treated for 7 days with the indicated hormones, either in the absence or presence of 100 nM RU486, then infected as in Figure 1. The results show pooled data from at least four independent experiments, with two to three bbPBMC donors each, for a total of 18 independent donors for –RU486, including *n* = 7 from Figure 1 to 11 independent donors for +RU486, performed in parallel. Infection was plotted relative to the vehicle controls in the presence of virus set to 100%. (B) A representative western blot indicating GR and GAPDH protein levels from matched PBMC samples at Day 0, 2, 7, and 12 post‐treatment with the indicated hormones. (C) Western blots were scanned and quantified for relative GR levels by calculating integrated density values from *n* = 7 independent donors. Statistical comparisons were carried out using a two‐way ANOVA with multiple comparisons post‐tests, indicated by stars (*), or a Wilcoxon test, indicated by letters (a–d). Stars or letters above bars indicate significance for the comparison indicated by lines, with ****/a, ***/b, **/c and */d indicating *p* < .0001, *p* < .001, *p* < .01, and *p* < .05 respectively

To assess whether GR mediates the increase in HIV‐1 replication in bbPBMCs treated with luteal phase E2+P4 or MPA, bbPBMCs were co‐treated with the GR/PR antagonist RU486 for 7 days prior to infection. As before, HIV‐1 infection was increased in MPA‐ and luteal phase E2+P4‐treated bbPBMCs (Figure 2A). Co‐treatment with RU486 significantly reduced HIV‐1 replication in MPA‐treated bbPBMCs, but no significant effect was detected for luteal or follicular phase E2+P4 (Figure 2A). Similar results were obtained in TZM‐bl cells, where knockdown of the GR significantly decreased the effect of MPA but not luteal phase hormones (data not shown).

When further investigating the role of the GR, we found that treatment with MPA, but not luteal phase E2+P4, decreased GR protein levels in PBMCs treated for various durations (Figure 2B,C). Since the GR protein is turned over upon activation by ligands,^70^, ^75^, ^76^, ^77^, ^78^, ^79^, ^80^ these results suggest that while the effects of MPA‐induced HIV‐1 infection are mediated by the GR, this is unlikely to be a mechanism by which luteal phase E2+P4 increases HIV‐1 replication in PBMCs.

### 3.3 MPA but not luteal or follicular phase E2+P4 increases CD4/CD8 ratios and CCR5 and CD69 expression on CD4+ T cells

**FIGURE 3:**
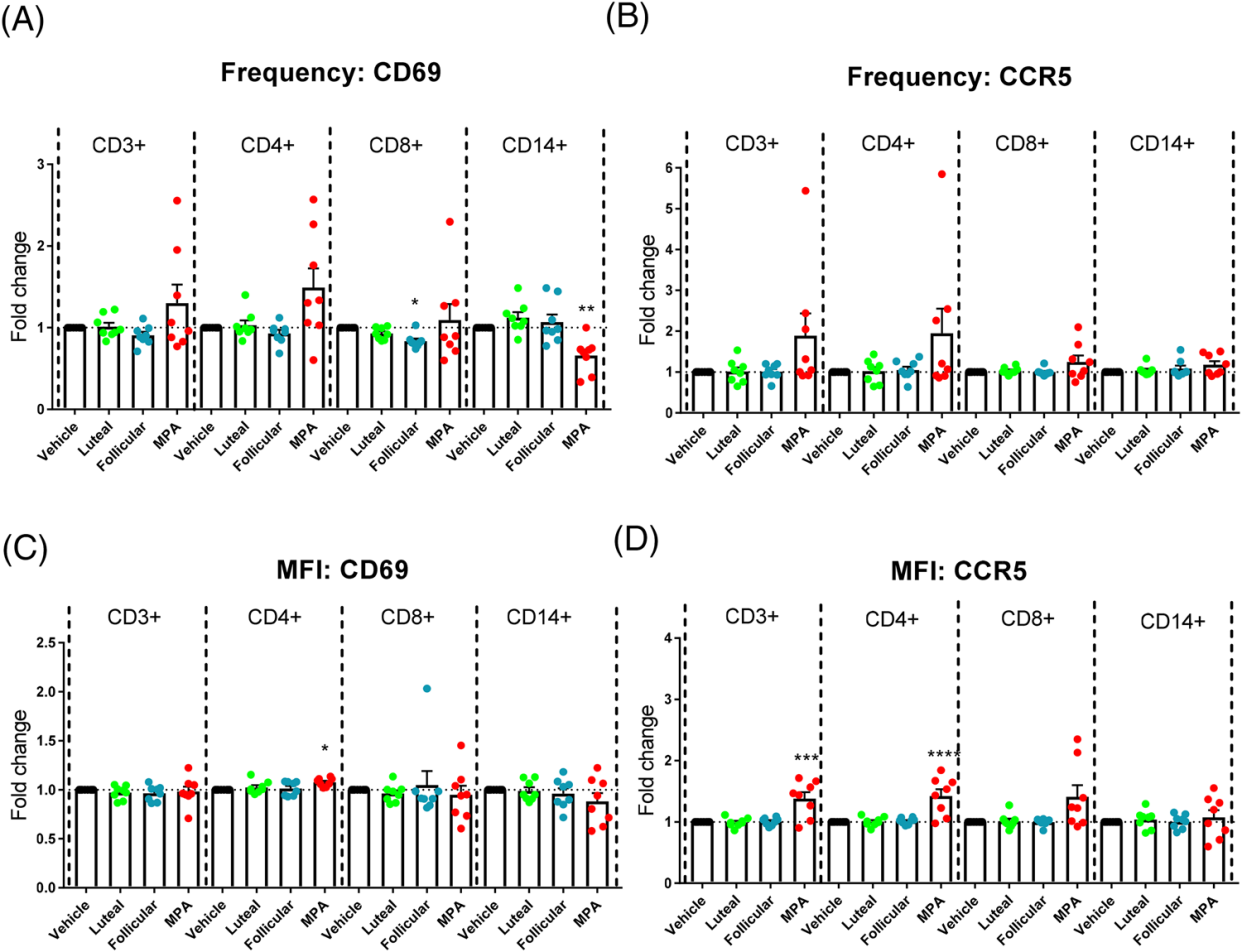
MPA, unlike luteal or follicular phase E2+P4, increases expression of CCR5 on CD3+ and CD4+ T cells and expression of CD69 on CD4+ T cells. bbPBMCs were treated for 7 days with the indicated hormones then stained with conjugated antibodies specific for the relevant markers, fixed and then sorted by flow cytometry. Samples were gated as shown in Supplementary Figure S1. The results are pooled from 2 independent experiments, with three to four donors each, for a total of seven to eight independent donors. Data are plotted as frequency (A, B) or median fluorescent intensity (MFI) (C, D) relative to the vehicle control set to 1. Statistical comparisons were carried out using a parametric one‐way ANOVA with Dunnett's multiple comparisons post‐test or paired *t*‐tests or a non‐parametric Kruskal‐Wallis test with Dunn's multiple comparisons post‐test or paired *t*‐tests. Stars above bars indicate significance compared to own vehicle control, unless between groups as shown by lines, with ****, ***, ** and * indicating *p* < .0001, *p* < .001, *p* < .01, and *p* < .05 respectively. Some data (Vehicle vs. MPA) has previously been published in a different context^40^ but are included here in the context of comparison to luteal and follicular phase data

Next we assessed the effects of these ligands on the frequency and expression (MFI) of immunological cell surface markers relevant to HIV‐1 infection: the host HIV‐1 co‐receptor CCR5 and the activation marker CD69, after 7 days’ treatment in CD3+, CD4+ and CD8+ T cells and CD14+ monocytes. Treatment with MPA appeared to increase both the frequency of CD69‐expressing (Figure 3A) and CCR5‐expressing (Figure 3B) CD3+ and CD4+ T cells, although not significantly. However, the expression of CD69 on CD4+ T cells (Figure 3C) and CCR5 on CD3+ and CD4+ T cells (Figure 3D) was significantly increased in MPA‐treated bbPBMCs compared to the vehicle control. There was also a significant decrease in the frequency of CD69‐expressing CD14+ monocytes in MPA‐treated PBMCs (Figure 3A). Treatment with luteal phase E2+P4 did not detectably change the frequency or MFI of CD69 or CCR5 in any cell type (Figure 3A–D). However, follicular phase E2+P4 significantly decreased the frequency of CD69‐expressing CD8+ T cells (Figure 3A).

**FIGURE 4:**
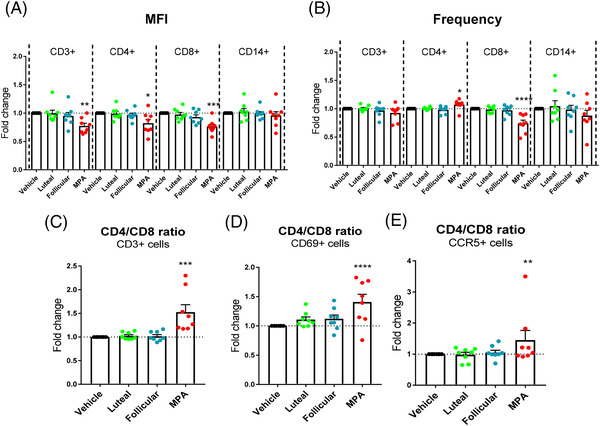
MPA but not luteal or follicular phase E2+P4 increases the CD4/CD8 ratio in CD3+, CD69+ and CCR5+ cells. bbPBMCs were treated and stained as indicated in Figure 3. Data are plotted as MFI (A), frequency (B) or CD4/CD8 ratio in CD3+ (C), CD69+ (D) or CCR5+ (E) cells relative to the vehicle control set to 1. Statistical comparisons were carried out using a parametric one‐way ANOVA with Dunnett's multiple comparisons post‐test or a non‐parametric Kruskal‐Wallis test with Dunn's multiple comparisons post‐test. Stars above bars indicate significance compared to own vehicle control, with ****, ***, ** and * indicating *p* < .0001, *p* < .001, *p* < .01 and *p* < .05 respectively. Some data (Vehicle vs. MPA) has previously been published in a different context^40^ but are included here in the context of comparison to luteal and follicular phase data

Since CD8+ T cells are cytotoxic towards HIV‐1, the CD4/CD8 ratio is an important parameter to consider, indicative of the balance between frequency of infectable cells (CD4+) and cells combating infection (CD8+). Despite the decrease in expression of CD3, CD4 and CD8 in MPA‐treated cells (Figure 4A), there was an increase in the frequency of CD4+ T cells, which together with the decreased frequency of CD8+ T cells (Figure 4B) revealed a significant increase in the CD4/CD8 ratio in MPA‐treated CD3+ T cells (Figure 4C) and in the subset of both CD69+ cells (Figure 4D) and CCR5+ cells (Figure 4E). No differences were detected for CD4 or CD8 frequency or expression, or the CD4/CD8 ratio, in cells treated with luteal or follicular phase E2+P4 (Figure 4A–E). Supplementary Tables S3 and S4 show the mean and standard error of the mean (SEM) for these frequency and expression data, respectively.

### 3.4 DMPA‐IM users exhibit increased frequency of CCR5+ T cells, unlike naturally cycling women in the luteal or follicular phase

We next investigated the frequency and expression of CCR5 and CD69 by flow cytometry in hPBMCs from a small cohort of naturally cycling women or those on DMPA‐IM (median age 43). PBMCs were isolated from a small cohort of female donors (nine DMPA‐IM users, seven women in the luteal phase, five women in the follicular phase) classified according to their prior recent DMPA‐IM use or with menstrual cycle status determined by their LH, FSH, E2 and P4 levels (Table S1‐2). Serum E2 levels were significantly lower in DMPA‐IM users (137.8 ± 90.14 pM) compared to women in the luteal phase (479.3 ± 268.5 pM), while serum P4 levels were significantly higher in women in the luteal phase (22.2 ± 6.148 nM) compared to either DMPA‐IM users (2.1 ± 1.39 nM) or women in the follicular phase (1.7 ± 2.001 nM) (Figure S5). The E2 levels used for treated bbPBMCs were within the range of these serum E2 levels, whereas serum P4 levels were about twice the concentration used for treated bbPBMCs, which were within the physiological range of 1–86 nM.^15^, ^21^, ^22^, ^23^

**FIGURE 5:**
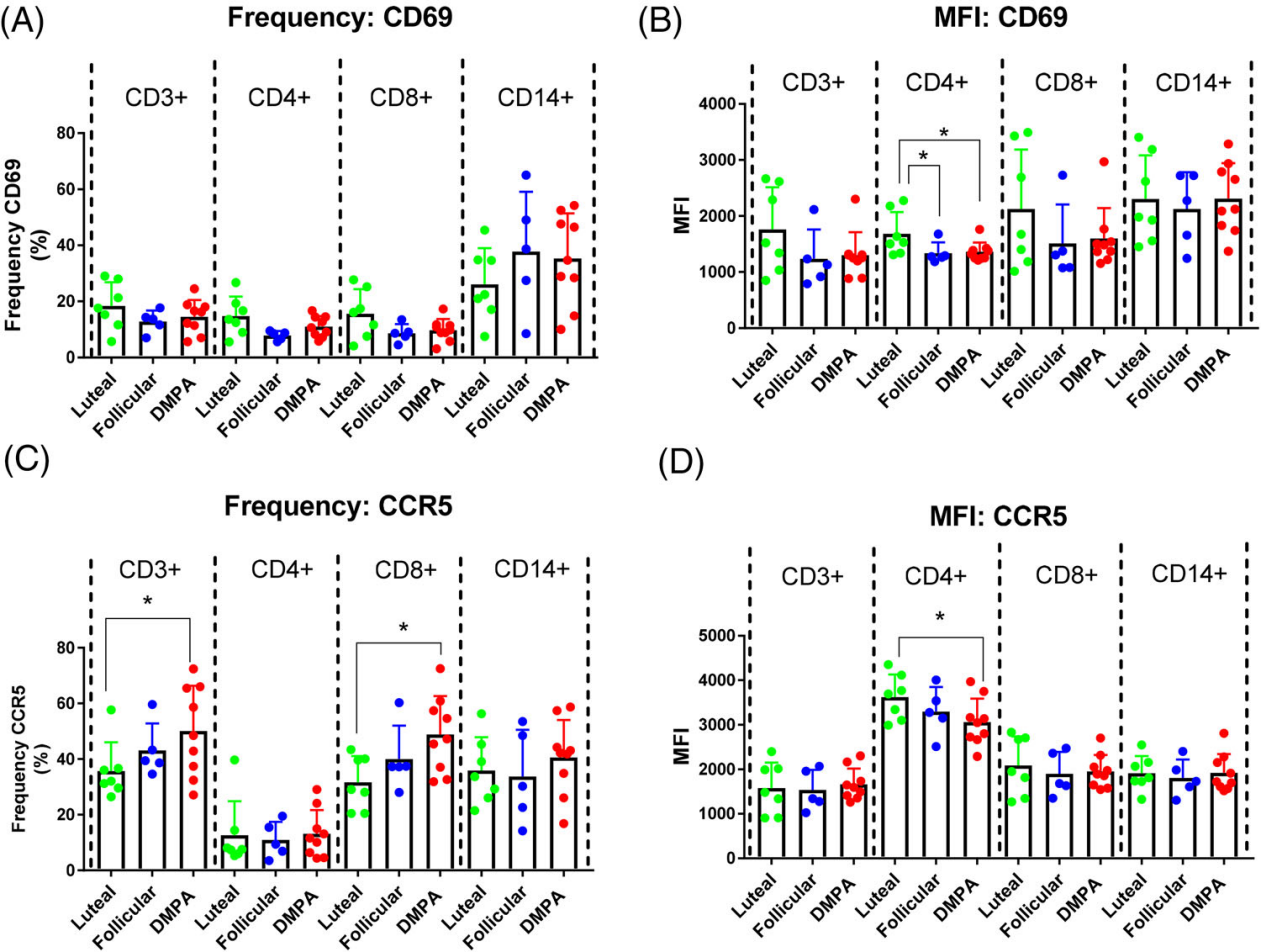
The frequency of CCR5‐expressing cells is higher in CD3+ and CD8+ T cells of intramuscular depo‐medroxyprogesterone acetate (DMPA‐IM) users compared to women in the luteal phase. Archived ex vivo PBMCs taken at time of hysterectomy (hPBMCs) from women in the luteal phase (*n* = 7) or follicular phase (*n* = 5) or DMPA‐IM users (*n* = 9) were thawed and stained for the indicated markers by flow cytometry. Data are plotted as non‐normalized raw values of frequency (A, C) or MFI (B, D) of the indicated marker/s. Statistical comparisons were carried out using a one‐way ANOVA with Tukey's multiple comparisons post‐test or unpaired t tests or Mann Whitney t tests. Stars show significance between the indicated groups, with ** and * indicating *p* < .01 and *p* < .05 respectively

The frequency of CD69‐expressing CD4+ T cells appeared to be higher in both women in the luteal phase and DMPA‐IM users compared to women in the follicular phase (Figure 5A). In line with this, the expression of CD69 on CD4+ T cells was significantly higher in hPBMCs from women in the luteal phase compared to both the follicular phase and DMPA‐IM users (Figure 5B). The frequency of CD69‐expressing CD8+ T cells appeared to be higher in women in the luteal phase compared to both women in the follicular phase and DMPA‐IM users (Figure 5A). The frequency of CCR5‐expressing CD3+ and CD8+ T cells was higher in DMPA‐IM users than women in the luteal phase (Figure 5C), but the expression of CCR5 on CD4+ T cells was lower (Figure 5D). The frequency of CCR5‐expressing CD3+ T cells appeared to be higher in women in the follicular compared to the luteal phase (Figure 5C). No significant differences in the frequency of CD3+, CD4+ or CD8+ T cells were detected in hPBMCs, but there appeared to be a higher frequency in CD14+ monocytes in DMPA‐IM users compared to women in the luteal phase (Figure S6A). However, there was significantly higher expression of cell surface CD3 and CD8 in hPBMCs from women in the luteal phase compared to DMPA‐IM users (Figure S6B). No significant differences were detected between groups in the CD4/CD8 ratio in CD3+ (Figure S6C), CD69+ (Figure S6D) or CCR5+ (Figure S6E). For the mean and SEM values of the frequency and expression data, see Supplementary Tables S5 and S6, respectively.

## 4 DISCUSSION

In this study we investigated the effects of MPA compared to follicular or luteal phase E2+P4 levels on HIV‐1 infection and cell surface markers in PBMCs from blood bank donors (bbPBMCs) treated with ligands in vitro, as well as from naturally cycling women or DMPA‐IM users (hPBMCs). Our results indicate that while in vitro treatment with both MPA and luteal phase E2+P4 increases HIV‐1 infection in bbPBMCs, the underlying mechanism appears to be different, with MPA increasing CCR5 and CD69 expression and the CD4/CD8 ratio in HIV‐1 target cells, unlike luteal phase E2+P4. The effects of MPA increasing CCR5 and CD69 are consistent with other in vitro and in vivo studies in PBMCs.^40^, ^43^, ^81^, ^82^

Our results also showed that in vitro HIV‐1 infection in response to MPA treatment in bbPBMCs most likely involves the GR. This is consistent with the partial agonist property of MPA for the GR^57^, ^58^, ^59^ and supports the mechanism that MPA‐induced GR activation may regulate genes relevant to HIV‐1 infection.^50^, ^83^

It is unclear from our data what mechanism might explain the increase in HIV‐1 infection in response to luteal phase E2+P4. Our in vitro results suggest that the GR is not involved in the luteal phase response. However, since RU486 treatment also appeared to decrease infection in luteal phase E2+P4‐treated PBMCs, we cannot exclude the possibility that a significant effect might be observed with an increased number of PBMC donors. While P4 can also act as a partial agonist for the GR,^57^, ^58^ comparing the low concentrations used to mimic luteal or follicular phase in these experiments (1–10 nM) with the K*_d_* of P4 for the GR (215 nM)^58^ suggests that these P4 concentrations are unlikely to result in a similar response to that of MPA (K*_d_* for the GR of 10.8 nM)^58^ via the GR. P4 binding to the GR may therefore only be relevant at higher P4 concentrations,^84^, ^85^, ^86^, ^87^ such as pregnancy where P4 is immunosuppressive in order to promote fetal tolerance.^88^, ^89^ Since we and others^61^, ^90^ have not detected classical nuclear PR in PBMCs, we could speculate that the P4 in the luteal phase treatment might be acting via the membrane PR (mPR). mPR is a G protein‐coupled receptor expressed on T cells^90^, ^91^, ^92^ that has a higher affinity for P4 than progestins,^93^ suggesting that it would not be affected by MPA or RU486. Since the concentration of E2 used in our study was identical for in vitro luteal and follicular phase E2+P4, the difference we observed in HIV‐1 infection is likely due to the different P4 concentrations.

It is likely that other immune cell types are involved in the luteal phase response we observed in vitro in bbPBMCs. A recent study showed decreases in B cells and conventional dendritic cells in the luteal compared to the follicular phase in pig‐tailed macaques,^13^ suggesting possible B cell involvement in our results. The luteal phase mechanism could also involve changes in immune cell function in response to activating stimuli and/or other cell surface markers not examined in our study. For example, increases in PD1, TNFα and CD38 but decreased FoxP3 were observed in the luteal phase compared to the follicular phase in women and/or pig‐tailed macaques.^13^ These cell types and markers warrant further investigation in our in vitro model.

Our results showing that incubation of bbPBMCs with follicular phase hormones had no effect on HIV‐1 infection in vitro are consistent with studies in naturally cycling macaques^7^, ^11^, ^12^ or macaques treated with a P4‐releasing capsule^24^ but not with one study in activated human PBMCs treated in vitro with 1 nM P4 + 100 pM E2.^16^ The latter results could be different because our bbPBMCs were non‐activated and we used different E2+P4 concentrations to mimic the follicular phase.

Our results in hPBMCs from naturally‐cycling women or women on DMPA‐IM suggest distinct differences between groups and an effect for DMPA‐IM involving increased frequency of CCR5+ T cells for DMPA‐IM users. However, while some of the hPBMC flow cytometry results are consistent with previous studies in PBMCs,^94^, ^95^ our results are not identical for the bbPBMC and hPBMC models. The in vitro bbPBMC model informs on the direct effects of E2+P4 on immune markers, while the in vivo hPBMC model likely reflects both direct and indirect effects of E2, P4, LH, FSH and other hormones on multiple physiological processes. Differences in E2 and P4 levels may also in part contribute to differences observed for these PBMC models. Nevertheless, the PBMC models both show differences in CD69 and CCR5 MFI and/or frequency between MPA and the luteal phase, indicating that these markers are relevant and providing further support that differences in immune marker expression exist between DMPA‐IM users and women in the luteal phase. Future work investigating whether CCR5 or CD69 protein responses to MPA and/or luteal phase E2+P4 are GR‐mediated would be useful to compare to our previous result showing that MPA‐increased CD4 and CCR5 mRNA levels were decreased with RU486 treatment.^40^

It is likely that MPA concentrations play an important role in effects on immune cells markers and HIV infection. We have previously shown that MPA dose‐dependently increases in vitro HIV‐1 infection in PBMCs, TZM‐bl cells and cervical explants, and that treatment with MPA at levels similar to peak serum concentrations in DMPA‐IM users increases CCR5 levels in these models.^40^, ^41^ Serum MPA levels rise sharply in the first 2 weeks post‐injection, after which they gradually decline over the next 3 months.^49^, ^51^, ^96^ Therefore, inter‐individual differences in MPA concentrations are likely to be influencing our in vivo results in hPBMCs. This is supported by our observation that a negative correlation exists between self‐reported time post‐injection in DMPA‐IM users and CCR5 frequency (Figure S7A, B) and CD4/CD8 ratios in T cells (Figure S7C). We therefore could speculate that if our hPBMCs were sampled at the time of peak serum MPA levels, we might have observed increases in CCR5 and CD69 MFI similar to the bbPBMC results. The in vivo hPBMC data has limited power due to low sample sizes but lays the groundwork for a larger high‐powered clinical study. In addition, the bbPBMC data are limited by treatment with only single E2+P4 concentrations to mimic the luteal and follicular phases and the absence of other hormones to better represent the endogenous hormone environment.

Despite several limitations, this is, to our knowledge, the first study comparing in vitro HIV‐1 infection and key immune markers between luteal phase, follicular phase and MPA in PBMCs, as well as a parallel study in PBMCs from women on DMPA‐IM or naturally‐cycling women. Overall, our results show that while both luteal phase E2+P4 and MPA increase HIV‐1 in PBMCs, CCR5 levels and target cell activation status in PBMCs are different between the luteal phase and MPA, for both in vitro treatment with ligands and for in vivo exposure, suggesting that HIV‐1 infection occurs via different mechanisms for women in the luteal phase and DMPA‐IM users.

## CONFLICT OF INTEREST

The authors declare no conflict of interest.

## Supporting information

Supporting Information

## Data Availability

All the data is in the manuscript. Additional raw data is available upon request from the authors.
