# Supplementary material for: Increased HIV‐1 infection in PBMCs treated in vitro with menstrual cycle phase hormones or medroxyprogesterone acetate likely occurs via different mechanisms
Source: Am J Reprod Immunol. 2022 Nov 2;88(6):e13643. doi: 10.1111/aji.13643 (PMC9884997; doi:10.1111/aji.13643)
Supplement: Supplementary file 1 — Supporting Information [file AJI-88-0-s001.docx]

**Supplementary information**

Increased HIV-1 infection in PBMCs treated *in vitro* with menstrual cycle phase hormones or medroxyprogesterone acetate likely occurs via different mechanisms

Alexis J. Bick, Chanel Avenant, Michele Tomasicchio, Zephne van der Spuy, Janet P. Hapgood

CD4

**Figure S1. Gating strategy for flow cytometry in PBMCs.** Cells were gated as monocytes or lymphocytes depending on size and granularity. Single cells were selected and gated as CD14+ or CD3+ based on FMO controls (data not shown). CD3+ cells were further categorized as CD4+ or CD8+ based on FMO controls. For all cell types (CD3+, CD4+, CD8+, CD14+), cells were further gated as CCR5+, CD69+ or CD25+ in select experiments, based on FMO controls. A representative PBMC donor is shown.

**Figure S2.** **Renilla luciferase activity increases after addition of HIV_BaL compared to virus control in the absence or presence of hormones mimicking the menstrual cycle phases or MPA.** bbPBMCs were treated and infected as in Fig. 1. The results show pooled data from at least 3 independent experiments, with 2-3 bbPBMC donors each, for a total of 7 independent donors. Infection was plotted relative to each day’s vehicle control plus virus set to 100%. Statistical comparisons were carried out using a two-way ANOVA with Tukey’s multiple comparisons post test. Significance is indicated by lines between samples with **** indicating p<0.0001.

**Figure S3. Higher concentrations of P4 do not further increase HIV-1 infection compared to lower concentrations.** bbPBMCs were treated with the indicated ligands for 2 days and then infected as in Fig 1. The results show pooled data from at least 3 independent experiments, with 2-3 bbPBMC donors each, for a total of 10 independent donors. Infection was plotted relative to the vehicle control plus virus set to 100%. Statistical comparisons were carried out using Mann Whitney t tests. Stars above bars indicate significance compared to the vehicle control, or between the groups indicated by a line, with ****, ** and * indicating p<0.0001, p<0.01 and p<0.05 respectively. Luteal: 10 nM P4 + 400 pM E2. MPA: 100 nM MPA.

**Figure S4. PR protein is not detectably induced in bbPBMCs following treatment with luteal phase E2+P4.** bbPBMCs (8 million) from 3 independent donors (#5209, #6591, #7186) were treated with hormones representing the luteal phase (10 nM P4 + 400 pM E2) for 0, 2 or 7 days then pelleted and harvested for total protein using TAPS buffer. Lysates were analyzed for PR protein levels by western blotting using GAPDH as a loading control. The GAPDH image was cropped to exclude a lane for an unrelated PR-negative sample.

**Figure S5. Endogenous hormone levels in hPBMCs from women in the luteal phase or follicular phase or DMPA-IM users.** LH (A), FSH (B), E2 (C) and P4 (D) levels (Table S1) were measured at the South African NHLS by electrochemiluminescence immunoassay for most donors, while information was unavailable for some donors (Table S1). The concentrations of E2 and P4 used for the stimulation experiments are indicated by dotted lines (C, D). Statistical comparisons were carried out using a parametric one-way ANOVA with Tukey’s post test (B) or non-parametric Kruskal-Wallis test with Dunn’s post test (A, C-D). Stars indicate significance for the comparisons indicated by lines, with ** and * indicating p<0.01 and p<0.05 respectively.

**Figure S6. No differences in CD4/CD8 ratios occur between women in the luteal phase and DMPA-IM users.** Archived *ex vivo* hPBMCs from women in the luteal phase (n=7) or follicular phase (n=5) or DMPA-IM users (n=9) were thawed and stained for the indicated markers by flow cytometry. Data are plotted as non-normalized raw values of frequency (A) or MFI (B) or CD4/CD8 ratio in CD3+ (C), CD69+ (D) or CCR5+ (E) cells. Statistical comparisons were carried out using a one-way ANOVA with Tukey’s multiple comparisons post-test or unpaired t tests. Stars show significance between the indicated groups, with ** and * indicating p<0.01 and p<0.05 respectively.

**Figure S7. CCR5 frequency in CD3+ and CD4+ cells and the CD4/CD8 ratio correlate with time post-injection in DMPA-IM users.** The frequency of CCR5 in CD3+ (A) or CD4+ (B) cells and the CD4/CD8 ratio (C) were plotted against time since self-reported DMPA injection in archived *ex vivo* hPBMCs from DMPA-IM users (n=5) (Table S1) and analyzed for Pearson’s correlation and coefficient of determination (R^2^). * indicates significance with p<0.05.

**Supplementary tables**

**Table S1 Clinical information from archived *ex vivo* hPBMC donors**

| **Date of collection** | **Patient number** | **Age** | **HIV** | **HPV** | **HSV-1** | **HSV-2** | **LH (IU/l)** | **FSH (IU/l)** | **E2 (pM)** | **P4 (nM)** | **Date of last contraception** |
| --- | --- | --- | --- | --- | --- | --- | --- | --- | --- | --- | --- |
| **FOLLICULAR PHASE** | | | | | | | | | | | |
| 23 Mar 2017 | PROS0005 | 50 | Negative | Negative | Positive | Positive | 1.9 | 4.6 | 95 | 0.3 | Oral contraceptive (last dose Nov 2016) |
| 2 Feb 2016 | PROT0020 | 38 | Negative | Negative | Positive | Positive | 1.9 | 2.4 | 1148 | 3.3 | N/A |
| 10 Mar 2017 | PROT0073 | 44 | Negative | Negative | Positive | Negative | 22.6 | 12.2 | 922 | 0.3 | Petogen Injection (last dose 2009) |
| 2 May 2017 | PROT0078 | 45 | Negative | Negative | Positive | Negative | 6.8 | 7.9 | 163 | <0.2 | Petogen Injection (last dose Jun 2016) |
| 24 Aug 2017 | PROT0091 | 37 | Negative | Negative | Positive | Negative | 2.3 | 8.3 | 81 | 4.4 | Petogen Injection (last dose 2016) |
| **LUTEAL PHASE** | | | | | | | | | | | |
| 19 Jul 2018 | PROG137 | 33 | Negative | Negative | Positive | Positive | 3.7 | 3.6 | 386 | 17.7 | N/A |
| 16 Nov 2017 | PROG124 | 40 | Negative | Negative | Positive | Negative | 4.2 | 1.9 | 365 | 25.6 | N/A |
| 24 May 2018 | PROG133 | 39 | Negative | Negative | Positive | Negative | 5.1 | 3.5 | 1036 | 26.5 | N/A |
| 18 Aug 2016 | PROG097 | 34 | Negative | Negative | Positive | Negative | 7.6 | 3.9 | 384 | 32.1 | N/A |
| 13 Apr 2017 | PROS007 | 50 | Negative | Negative | Positive | Positive | 11 | 9.7 | 315 | 15.9 | Oral contraceptive |
| 12 Jul 2016 | PROT058 | 39 | Negative | Negative | Positive | Positive | 3.4 | 2.9 | 260 | 16 | Nuristerate Injection (last dose 2004) |
| 28 Jan 2019 | PROG155 | 39 | Negative | ND | ND | ND | 9.6 | 6.9 | 609 | 21.6 | ND |
| **DMPA-IM USERS** | | | | | | | | | | | |
| 29 Sep 2017 | PROT092* | 44 | Negative | Negative | Positive | Negative | 5.7 | 9.7 | 85 | <0.2 | Depo Provera Injection (last dose late Jul 2017) |
| 16 Mar 2016 | PROT030* | 46 | Negative | Negative | Positive | Positive | 0.6 | 3.7 | ND | 2.6 | Depo Provera Injection (last dose 25 Feb 2016) |
| 28 Jun 2016 | PROT053* | 36 | Negative | Negative | Positive | Positive | 4.3 | 8.9 | 100 | 0.8 | Depo Provera/Nordette (last dose early Apr 2016 |
| 5 Apr 2016 | PROT036 | 51 | Negative | Negative | Positive | Positive | 4.6 | 4 | 162 | ND | Depo Provera Injection (last dose Dec 2015) |
| 22 Apr 2016 | PROT038* | 43 | Negative | Negative | Positive | Positive | ND | ND | ND | ND | Depo Provera (unknown last dose) |
| 1 Sep 2016 | PROG099 | 40 | Negative | Negative | Positive | Negative | 5.3 | 6 | 289 | 2.2 | Petogen Injection (last dose May 2016) |
| 2 May 2017 | PROT077* | 45 | Negative | Negative | Positive | Negative | 0.7 | 5.3 | 27 | 2.8 | Petogen Injection (last dose 27 Jan 2017) |
| 22 Apr 2016 | PROT039 | 49 | Negative | Negative | Positive | Positive | ND | ND | ND | ND | Petogen Injection (last dose Jan 2016) |
| 2 Mar 2017 | PROS003 | 43 | Negative | Negative | Positive | Positive | 2.8 | 4.2 | 164 | 4 | Petogen Injection (unknown last dose) |

* patient data used for Pearson correlations

**Table S2 Summary of clinical information from archived *ex vivo* hPBMC donors**

|  |  |  |  | **p value#** | | |
| --- | --- | --- | --- | --- | --- | --- |
|  | **Follicular** | **Luteal** | **DMPA-IM** | **Foll vs Lut** | **Lut vs DMPA** | **Foll vs DMPA** |
| **n** | 5 | 7 | 9 |  |  |  |
| **Ave age (years)** | 42,8 | 39,1 | 44,1 | 0,4473 | 0,1528 | 0,888 |
| **HIV+** | 0/5 | 0/7 | 0/9 |  |  |  |
| **HPV+** | 0/5 | 0/6* | 0/9 |  |  |  |
| **HSV-1+** | 5/5 | 6/6* | 9/9 |  |  |  |
| **HSV-2+** | 2/5 | 3/6* | 2/3 |  |  |  |
| **Ave LH (IU/l) [range]** | 7,1 [1,9-22,6] | 6,4 [3,4-11] | 3,4 [0,6-5,7] | 0,9809 | 0,4621 | >0,9999 |
| **Ave FSH (IU/l) [range]** | 7,1 [2,4-12,2] | 4,6 [1,9-9,7] | 6 [3,7-9,7] | 0,3461 | 0,6707 | 0,795 |
| **Ave E2 (pM) [range]** | 481,8 [81-1148] | 479,3 [260-1036] | 137,3 [27-289] | 0,7484 | **0,0347** | 0,6829 |
| **Ave P4 (nM) [range]** | 2,1 [0,2-4,4] | 22,2 [15,9-32,1] | 2,5 [0,2-2,8] | **0,0087** | **0,0096** | >0,9999 |

*1 donor not determined

#One way ANOVA + Tukey's post test (Age, FSH) or Kruskal Wallis + Dunn's post test (LH, E2, P4)

**Table S3. Frequencies of total, CD69+ and CCR5+ cells expressing the markers CD3, CD4, CD8 or CD14 in stimulated bbPBMCs.**

| **Immune cell phenotype:** | **Vehicle** | | **Luteal** | | **Follicular** | | **MPA** | |
| --- | --- | --- | --- | --- | --- | --- | --- | --- |
| **Frequency** | **Mean** | **SEM** | **Mean** | **SEM** | **Mean** | **SEM** | **Mean** | **SEM** |
| **CD3+ (% total)** | 79,70 | 3,57 | 79,98 | 3,10 | 76,71 | 3,75 | 73,13 | 3,90 |
| **CD3+CD69+** | 5,63 | 0,96 | 5,73 | 1,07 | 5,05 | 0,88 | 6,16 | 0,53 |
| **CD3+CCR5+** | 23,44 | 5,23 | 24,66 | 5,61 | 23,36 | 4,81 | 32,24 | 4,06 |
| **CD4+ (% total)** | 63,43 | 4,63 | 63,84 | 4,75 | 62,06 | 4,57 | 67,43 | 4,13 |
| **CD4+CD69+** | 6,84 | 0,92 | 6,98 | 0,92 | 6,16 | 0,52 | 9,44 | 1,32 |
| **CD4+CCR5+** | 21,03 | 4,47 | 22,68 | 5,27 | 21,71 | 4,32 | 28,98 | 3,55 |
| **CD8+ (% total)** | 27,35 | 4,03 | 27,13 | 4,29 | 26,83 | 4,38 | 21,37 | 4,37 |
| **CD8+CD69+** | 6,38 | 0,97 | 5,98 | 1,03 | 5,31 | 0,78 | 6,33 | 0,82 |
| **CD8+CCR5+** | 47,55 | 8,44 | 48,91 | 8,64 | 47,24 | 8,12 | 53,15 | 6,57 |
| **CD14+ (% total)** | 36,81 | 13,52 | 34,14 | 12,43 | 35,38 | 12,98 | 35,05 | 13,19 |
| **CD14+CD69+** | 29,98 | 1,73 | 33,11 | 1,32 | 31,71 | 2,79 | 19,19 | 1,81 |
| **CD14+CCR5+** | 72,06 | 8,93 | 72,91 | 7,75 | 75,05 | 7,40 | 79,60 | 5,14 |
| **CD4/CD8 ratio (total)** | 3,16 | 0,94 | 3,23 | 0,93 | 3,18 | 0,90 | 4,84 | 1,28 |
| **CD4/CD8 ratio: CD69+** | 1,33 | 0,35 | 1,41 | 0,33 | 1,41 | 0,30 | 1,65 | 0,24 |
| **CD4/CD8 ratio: CCR5+** | 0,43 | 0,05 | 0,44 | 0,07 | 0,47 | 0,08 | 0,58 | 0,11 |

Mean and SEM are shown as percentages or CD4/CD8 ratios, not normalized to fold change, and were calculated from 8 independent donors. CD69+ and CCR5+ cells are frequencies of the cells expressing CD3, CD4, CD8 or CD14. Some data (Vehicle vs MPA) has previously been published (Maritz et al. 2018) but is included here in the context of comparison to luteal and follicular phase data.

**Table S4. Expression of CD69 or CCR5 in cells expressing the markers CD3, CD4, CD8 or CD14 in stimulated bbPBMCs.**

| **Immune cell phenotype:** | **Vehicle** | | **Luteal** | | **Follicular** | | **MPA** | |
| --- | --- | --- | --- | --- | --- | --- | --- | --- |
| **Expression** | **Mean** | **SEM** | **Mean** | **SEM** | **Mean** | **SEM** | **Mean** | **SEM** |
| **CD3** | 9039 | 476,5 | 8915 | 461,3 | 8535 | 602,2 | 6946 | 422,9 |
| **CD3+CD69+** | 4178 | 431,6 | 4056,88 | 451,6 | 4028,75 | 444,6 | 4023,5 | 340 |
| **CD3+CCR5+** | 2609,25 | 142,7 | 2579,63 | 128,5 | 2621,75 | 140,4 | 3551,38 | 245 |
| **CD4** | 8333 | 596,9 | 8241 | 659,4 | 8098 | 690 | 6659 | 358,9 |
| **CD4+CD69+** | 2170,75 | 234 | 2206,63 | 219 | 2196 | 242 | 2328,38 | 240,2 |
| **CD4+CCR5+** | 2496,38 | 104 | 2516,5 | 149,6 | 2539,25 | 140,3 | 3533,63 | 278,5 |
| **CD8** | 44095 | 8157 | 43658 | 8328 | 42219 | 8429 | 32038 | 5220 |
| **CD8+CD69+** | 4972,75 | 550,6 | 4770,13 | 524 | 5032,38 | 590,6 | 4499,5 | 318,6 |
| **CD8+CCR5+** | 1193,38 | 146,9 | 1197,5 | 141,6 | 1170,75 | 116,8 | 1650,5 | 263,5 |
| **CD14** | 2354 | 274,6 | 2432 | 339,9 | 2336 | 287,4 | 2221 | 309,2 |
| **CD14+CD69+** | 4202,75 | 122 | 4163,75 | 217,7 | 4069,5 | 302,2 | 3725 | 403,2 |
| **CD14+CCR5+** | 5203,88 | 1223 | 5345,88 | 1333 | 4970,75 | 1107 | 4803,25 | 1012 |

Mean and SEM represent the MFI, not normalized to fold change, of CD69 or CCR5 expressed on the indicated cells and were calculated from 8 independent donors. Some data (Vehicle vs MPA) has previously been published (Maritz et al. 2018) but is included here in the context of comparison to luteal and follicular phase data.

**Table S5 Frequencies of total, CD69+ and CCR5+ cells expressing the markers CD3, CD4, CD8 or CD14 in archived *ex vivo* hPBMCs.**

| **Immune cell phenotype:** | **Luteal** | | **Follicular** | | **MPA** | |
| --- | --- | --- | --- | --- | --- | --- |
| **Frequency** | **Mean** | **SEM** | **Mean** | **SEM** | **Mean** | **SEM** |
| **CD3+ (% total)** | 39,71 | 4,99 | 25,04 | 6,79 | 34,09 | 5,37 |
| **CD3+CD69+** | 18,36 | 3,20 | 12,86 | 1,76 | 14,49 | 2,00 |
| **CD3+CCR5+** | 35,69 | 3,90 | 43,10 | 4,35 | 50,09 | 5,42 |
| **CD4+ (% total)** | 47,23 | 2,60 | 53,66 | 3,72 | 49,33 | 3,90 |
| **CD4+CD69+** | 14,76 | 2,63 | 7,83 | 0,71 | 11,03 | 1,25 |
| **CD4+CCR5+** | 12,64 | 4,65 | 10,98 | 2,89 | 13,15 | 2,85 |
| **CD8+ (% total)** | 60,60 | 5,58 | 59,36 | 4,27 | 62,33 | 5,75 |
| **CD8+CD69+** | 15,67 | 3,29 | 8,66 | 1,45 | 9,72 | 1,32 |
| **CD8+CCR5+** | 31,61 | 3,59 | 40,04 | 5,38 | 48,84 | 4,59 |
| **CD14+ (% total)** | 27,67 | 6,88 | 28,46 | 5,72 | 45,49 | 6,62 |
| **CD14+CD69+** | 26,11 | 4,85 | 37,74 | 9,57 | 35,29 | 5,37 |
| **CD14+CCR5+** | 35,90 | 4,54 | 33,80 | 7,51 | 40,58 | 4,48 |
| **CD4/CD8 ratio (total)** | 0,83 | 0,10 | 0,93 | 0,12 | 0,90 | 0,16 |
| **CD4/CD8 ratio: CD69+** | 1,09 | 0,17 | 1,04 | 0,27 | 1,29 | 0,21 |
| **CD4/CD8 ratio: CCR5+** | 0,40 | 0,12 | 0,30 | 0,10 | 0,26 | 0,06 |

Mean and SEM are shown as percentages or CD4/CD8 ratios, not normalized to fold change, and were calculated from n=5 (Follicular), n=7 (Luteal) and n=9 (DMPA-IM users) donors. CD69+ and CCR5+ cells are frequencies of the cells expressing CD3, CD4, CD8 or CD14.

**Table S6 Expression of CD69 or CCR5 in cells expressing the markers CD3, CD4, CD8 or CD14 in archived *ex vivo* hPBMCs.**

| **Immune cell phenotype:** | **Luteal** | | **Follicular** | | **MPA** | |
| --- | --- | --- | --- | --- | --- | --- |
| **Expression** | **Mean** | **SEM** | **Mean** | **SEM** | **Mean** | **SEM** |
| **CD3** | 30158 | 1953 | 27069 | 2638 | 23050 | 1694 |
| **CD3+CD69+** | 1760 | 284,7 | 1238 | 232,7 | 1302 | 137,1 |
| **CD3+CCR5+** | 1577 | 217,7 | 1535 | 202,8 | 1663 | 118,8 |
| **CD4** | 2012 | 193,4 | 2060 | 85,3 | 1871 | 73,8 |
| **CD4+CD69+** | 1681 | 147,4 | 1339 | 86,5 | 1360 | 55,9 |
| **CD4+CCR5+** | 3624 | 192 | 3297 | 245,6 | 3061 | 176,3 |
| **CD8** | 7095 | 617,7 | 5369 | 856,5 | 3640 | 727,6 |
| **CD8+CD69+** | 2127 | 399,9 | 1513 | 309,7 | 1597 | 181,7 |
| **CD8+CCR5+** | 2085 | 248,1 | 1898 | 219,1 | 1952 | 124,2 |
| **CD14** | 2686 | 965,5 | 1985 | 873,8 | 2000 | 494,3 |
| **CD14+CD69+** | 2305 | 293,8 | 2125 | 294 | 2313 | 211,1 |
| **CD14+CCR5+** | 1911 | 146,8 | 1806 | 184,4 | 1919 | 141,8 |

Mean and SEM represent the MFI, not normalized to fold change, of CD69 and/or CCR5 expressed on the indicated cells and were calculated from n=5 (Follicular), n=7 (Luteal) and n=9 (DMPA-IM users) donors.
